# ‘Diagnostic terms are consequential’

**DOI:** 10.1192/bjb.2025.10187

**Published:** 2026-02

**Authors:** Rob Poole

**Affiliations:** Centre for Mental Health and Society, School of Health Sciences, Bangor University, UK

**Keywords:** Stigma and discrimination, personality disorders, exclusion, labelling, suicide

## Abstract

Patients find the term ‘borderline personality disorder’ offensive and, from a list of alternative labels, prefer ‘emotional intensity disorder’. It is suggested that any term will take on a pejorative connotation if professional attitudes do not change as well; and that this requires an alteration in the environment in which professionals operate. This should not look so strongly to compulsion to prevent suicide, but should allow therapeutic relationships to flourish. Blaming clinicians for incidents when they have few choices is counterproductive. The problem reflects a systemic impatience with patients who get better slowly or not at all.

In this edition of the *Bulletin*, Hayward et al^1^ show that patients would prefer a label that is less pejorative than ‘borderline personality disorder’ (BPD). There are a range of interesting findings in the paper, but the authors favour the term ‘emotional intensity disorder’, supported by a sort of plebiscite of a broad range of general adult psychiatric patients. Asking patients’ opinion is welcome, with some unexpected findings. The authors also argue that BPD should be grouped with the affective disorders. It is acknowledged that a new label might eventually become just as pejorative as the previous one, but it is hoped this might give an opportunity to change the stigma associated with the diagnosis. Suggestions of how this might be achieved are beyond the scope of the paper.

I am sympathetic with the objective of resetting attitudes to people with the diagnosis of BPD but, in my opinion, the paper addresses only the smaller part of the issue. When I was a medical student on psychiatric placement in the late 1970s, the term BPD was mentioned as an alternative to a range of highly stigmatising diagnoses in common use at the time, such as ‘hysterical personality disorder’, which, like the present-day use of BPD, was highly gendered. In 1984, Tarnopolsky and Berelowitz^2^ reported that just 25% of psychiatrists working at the Maudsley used the diagnosis. By 1992, it had taken on such a stigmatising connation that it was renamed ‘emotionally unstable personality disorder’ in ICD-10,^3^ a term that Hayward et al^1^ report as being just as offensive to psychiatric patients as BPD. There is little evidence that any benefit of a new semantic label or euphemism lasts very long, at least providing that the implication remains that it is the personality, the core of the self, that is wrong. The main attraction of ‘emotional intensity disorder’ seems to be that it avoids invoking personality and is not in general use. The question of whether BPD should be grouped with the affective disorders depends on whether it has any aetiologically or clinical features to justify this, and it conflates two separate issues: our understanding of personality problems on the one hand and the offensiveness of personality labels on the other.

The whole matter might fruitfully be reconsidered from a different angle. First, ‘borderline’ has no intrinsically offensive meaning and, as Hayward et al^1^ point out, is not even semantically coherent. It is how the term is used that is offensive. The term originates in pre-Second World War psychoanalytic practice and speculatively refers to the borderline between neurotic and psychotic disorders. Second, one of the analytic authorities on the subject, Kernberg,^4^ wrote about ‘borderline personality organisation’ (although he sometimes used the term ‘personality disorder’) which, while also speculative, is a nuance that was lost when the term was imported into mainstream psychiatric nosological systems. Third, while the full-blown BPD syndrome is recognisable, in reality the syndrome merges imperceptibly with other personality types. Furthermore, while personality traits can group together, forming categories, they do not necessarily do so. In life, the features can be extremely variable. The only real virtue of the BPD diagnosis is that it is said to predict what interventions may be unhelpful.

Everything suggests that BPD is not a discrete disorder analogous to a disease, but rather a developmental variant that can be extremely troublesome or not, depending on the circumstances in which the person finds themselves. Although many patients report an extremely difficult childhood, this is not invariable, and similar findings are found with other psychiatric diagnoses. Although there is a widespread belief that BPD is commonly due to childhood trauma, we do not really understand causality. The manifestation of BPD is dependent on context and does not lend itself to categorical diagnosis at all. This may be difficult for research, collating service data and the administration of benefits, but applying arbitrary inclusion criteria does violence to the clinical realities.

The main reason why BPD is regarded as pejorative by patients is our clinical behaviour, which is why renaming per se does not work as a stigma-eliminating tactic. All terms become stigmatising when patients and the general public come to realise that mental health professionals, mainly doctors and nurses, use them to dismiss people’s problems. These professions often use the term to signify that the patient’s behaviour is a moral problem rather than a medical one, and that it is untreatable. Beale^5^ has described how terminology is used to exclude people with real problems from access to services. This practice is not congruous with the evidence.^6^ The diagnosis of BPD is associated with a suicide rate that is similar to that of other psychiatric diagnoses, and it is a major risk factor for other mental health problems. Good results are reported for a range of interventions, mainly psychotherapies, although we are far from having a fully satisfactory response for all patients. In other words, the situation is similar to that appertaining to the diagnosis ‘schizophrenia’, another label that is contested and attracts a different type of stigma.

If we are to make progress in making better and more acceptable responses to people with the diagnosis of BPD, two things need to happen, which are quite separate from the continued and confusing roundabout of euphemism. First, our attitudes must change in a major way. This includes the recognition that professionals react to the sometimes difficult behaviour of these patients, often in ways that reiterate previous damaging experiences. We must find ways of helping clinicians to rise above this and to stop making judgemental decisions that exclude the patients or sequestrate them in secure settings.^7^ Second, policy-makers and managers must facilitate this. They have to recognise that attempts to control patients’ behaviour through compulsion often make things worse and raise risk. Clinical suicide prevention is mainly relational. Admission and compulsion will work only where the suicide risk is associated with a transient mental state that will resolve quickly. Even then, discharge is recognised as being a dangerous period. Governments must stop supporting legal measures that are known to be ineffective,^8^ using compulsion to compensate for the shortcomings of mental health services and take alternative measures to allow therapeutic relationships to flourish. Culpability for serious incidents should not rest solely with the last clinician to see the patient, but should include those who design a dysfunctional and ineffective system.

In summary, the problem of dealing with that group of patients who do not conveniently and promptly recover is the real issue, whether they are labelled BPD, ‘behavioural’ or ‘chronic’. I am not opposed in principle to a new terminology, but we should not let ourselves believe that its application will actually solve very much. We need services that resolve patients’ problems, not serve organisational needs.
